# Funding and inclusion in higher education institutions for students with disabilities

**DOI:** 10.4102/ajod.v8i0.336

**Published:** 2019-01-29

**Authors:** Desire Chiwandire, Louise Vincent

**Affiliations:** 1 Department of Political and International Studies, Rhodes University, South Africa

## Abstract

**Background:**

Historically, challenges faced by students with disabilities (SWDs) in accessing higher education institutions (HEIs) were attributed to limited public funding. The introduction of progressive funding models such as disability scholarships served to widen access to, and participation in, higher education for SWDs. However, recent years have seen these advances threatened by funding cuts and privatisation in higher education.

**Objectives:**

In this article, the funding mechanisms of selected developed and developing democratic countries including the United Kingdom, the United States, Canada, Australia, South Africa and India are described in order to gain an insight into how such mechanisms enhance access, equal participation, retention, success and equality of outcome for SWDs. The countries selected are often spoken about as exemplars of best practices in relation to widening access and opportunities for SWDs through government mandated funding mechanisms.

**Method:**

A critical literature review of the sample countries’ funding mechanisms governing SWDs in higher education and other relevant government documents; secondary academic literature on disability funding; online sources including University World News, University Affairs, newspaper articles, newsletters, literature from bodies such as the Organisation for Economic Co-operation and Development, Disabled World and Parliamentary Monitoring Group. Data were analysed using a theoretically derived directed qualitative content analysis.

**Results:**

Barriers which place SWDs at a substantial educational disadvantage compared to their non-disabled peers include bureaucratisation of application processes, cuts in disability funding, means-test requirements, minimal scholarships for supporting part-time and distance learning for SWDs and inadequate financial support to meet the day-to-day costs that arise as a result of disability.

**Conclusion:**

Although the steady increase of SWDs accessing HEIs of the sampled countries have been attributed to supportive disability funding policies, notable is the fact that these students are still confronted by insurmountable disability funding-oriented barriers. Thus, we recommend the need for these HEIs to address these challenges as a matter of urgency if they are to respect the rights of SWDs as well as provide them with an enabling environment to succeed academically.

## Introduction

In the 1980s and 1990s, segregation of students with disabilities (SWDs) from mainstream education was called into question by the inclusive schools movement (Simons & Masschelein 2005:217). These developments culminated in the 10 June 1994 Salamanca World Conference on Special Needs Education, which was attended by 300 participants from 92 countries and 25 international organisations. The gathering led to the signing of the *Salamanca Statement* on inclusive education, which stated that:

Regular schools with this inclusive orientation are the most effective means of combating discriminatory attitudes, creating welcoming communities, building an inclusive society and achieving education for all. (United Nations Educational, Scientific and Cultural Organisation [UNESCO] 1994:ix)

Following the *Salamanca Statement*, inclusive education has been widely accepted as a model for education (Maher 2009). Proponents see inclusive education as foundational to a more just society (Ainscow et al. 2004), because it obliges mainstream educational institutions to focus on increasing the participation and levels of attainment of SWDs as a historically marginalised group (Wolanin & Steele 2004:vii).

The Salamanca Conference highlighted the need to prioritise funding as a mechanism for fostering the inclusion of SWDs in higher education and encouraged the international community to:

give the highest policy and budgetary priority to improve their educational systems to enable them to include all children regardless of individuals’ differences and difficulties. (UNESCO 1994:ix)

Recent literature on funding mechanisms aimed at providing access to higher education for previously excluded constituencies has mainly taken the form of comparative case studies (Yang & McCall 2014). None of these studies has focused specifically on SWDs.^1^ Yet the latter have very particular individual funding needs depending on the type or severity of their disabilities. As a result of their varying and varied needs, SWDs differ with regard to the nature of the support that they require to function optimally in their everyday living and learning environments. In other words, while the overarching question of higher education funding is a burning one, SWDs cannot simply be addressed as part of the wider debate.

Moreover, as many scholars have pointed out, universities globally are experiencing a dramatic decline in government subsidies and an increase in student fees (De Jager & Gbadamosi 2010:254), which have negatively impacted on the functioning of universities. In 2014, McGrath and colleagues conducted a comparative study which analysed admission systems to higher education across 10 European Union member countries focusing on how these countries deal with the inclusion of SWDs (McGrath et al. 2014:5). The study found that cutbacks in these countries’ public funding resources reduced or negatively impacted on equity in admission to higher education for many students, including those with disabilities in these countries (McGrath et al. 2014:9).

What follows is an attempt to describe disability funding mechanisms and approaches employed in a sample of democratic states both in the global North and the global South to gain an insight into how such mechanisms do or do not enhance access, equal participation, retention, success and equality of outcome for SWDs. The sampled countries represent a diversity of global North and global South examples, all of which have in common that they are signatories to the 2006 United Nations Convention on the Rights of Disabled Persons (UNCRPD), which obliges member states to ‘ensure an inclusive education system at all levels’ including higher education and to provide reasonable accommodations (RAs) and appropriate support services tailored to individuals’ educational needs as a measure for ensuring that persons with disabilities (PWDs) can participate effectively in a free society.

These countries have each enacted non-discriminatory legislation and have put in place disability funding policies and mechanisms to facilitate the inclusion of SWDs in higher education institutions (HEIs), and to address their educational needs. The selected countries take a human rights-based approach, which prohibits discrimination against SWDs on the grounds of disability and this is also provided for, in most instances, in these countries’ constitutions. As democratic countries, the countries surveyed draw on a mix of principles of social justice, equality, widening participation, redress, equality of access, transformation, affirmative action principles, equality of opportunity and equity in their approach to promote access, retention and success in higher education for SWDs. Research on funding of disability in HEIs has taken a dichotomous form with the global North countries on the one hand being associated with having progressive policies and practices which enable them to meet best practices criteria (Eleweke & Rodda 2002). In contrast, developing countries or the global South on the other hand have been discussed as either struggling or failing to meet these criteria because seldom do these governments and HEIs prioritise expenditure on inclusive education-oriented initiatives, including funding of disability (Eleweke & Rodda 2002). South Africa and India fall into the category of global South countries. We compared South Africa and India with global North countries because the two countries share the characteristic of being ‘countries in transition’ while at the same time being regarded as evincing best practices regarding disability inclusion compared to other global South countries.

Drawing on a directed content analysis of policy and other documents, the article argues that availability of adequate funding to HEIs to support students, including those with disabilities, is central to the maintenance and enhancement of quality education (Daugherty et al. 2013:39). However, in the wake of government funding cuts, the privatisation of HEIs and overreliance on private sources to finance higher education, the trend is towards universities adopting selective inclusion of SWDs based on cost rather than on the principles of inclusion, access and equal chances of success for all.

## The study

Bowen (2009:27) describes document analysis as ‘a systematic procedure for reviewing or evaluating documents – both printed and electronic’. As a qualitative analytical research method, document analysis ‘requires that data be examined and interpreted to elicit meaning, gain understanding, and develop empirical knowledge’ (Bowen 2009:27). For the purpose of this study the following documents were reviewed: The United Nations Convention on the Rights of Persons with Disabilities (UNCRPD) and the 1994 Salamanca Conference documents; peer-reviewed articles on inclusive education and disability in higher education; reports on disability retrieved from the official websites of the World Health Organisation, the United Nations Educational, Scientific and Cultural Organisation (UNESCO); the Organisation for Economic Co-operation and Development (OECD); *Disabled World*; *University World News* and *Times Higher Education*.

Documents specific to the countries in the sample included:

- South Africa – *White Paper 3 on Higher Education Transformation*; the 1997 *White Paper on an Integrated National Disability Strategy*; the 1997 *Higher Education Act*; the 2001 *National Plan for Higher Education* and the 2001 *Education White Paper 6, Special Needs Education: Building an Inclusive Education and Training System* and national newspaper articles on the National Student Financial Aid Scheme (NSFAS).
- India – the *Equal Opportunities, Protection of Rights and Full Participation (PWD) Act* and the Higher Education for Persons with Special Needs (HEPSN) policy as retrieved from University Grants Commission official website.
- United States – Section 504 of the *Rehabilitation Act* of 1973 and the *Americans with Disabilities Act* of 1990 (ADA); various policy documents reporting on the country’s federal student aid programme, known as the Pell Grant Program; various OECD reports on the inclusion of SWDs in higher education and other reports from the US Department of Education.
- Australia – the document known as *A Fair Chance for All,* which aims to increase the enrolments of SWDs in Australian higher education as well as the 1992 *Disability Discrimination Act* (DDA) and various OECD reports on the inclusion of SWDs in higher education as well as national newspaper articles reporting on issues relating to the Disability Support Program (DSP) which is the Australian funding scheme for SWDs.
- Canada – the *Canadian Human Rights Act* (CHRA) and the *Canadian Charter of Rights and Freedoms* (CCRF); the Canada Student Grant for Services and Equipment for Students with Permanent Disabilities (CSGSESPD) and the Canadian Province of Ontario Bursary scheme for Students with Disabilities (BSWD) as well as OECD reports on the inclusion of SWDs in higher education retrieved from the Ontario Human Rights Commission website.
- United Kingdom – the *Equality Act*, 2010; OECD reports on the inclusion of SWDs in the UK higher education system; newspaper articles reporting on the country’s various disability funding schemes; documents relating to the Disabled Students’ Allowance (DSA) grant; Premium Funding and the Higher Education Funding Council for England (HEFCE) Student Opportunity funding.

## Findings

Table 1 summarised the findings of the study.

**TABLE 1:** Summary of findings.

Country	Supporting policies	Funding mechanisms	Coverage extent	Limitations	Current challenges
United Kingdom	*Equality Act* (2010)	Premium Funding, Student Opportunity funding	Assistive technology	No personal assistant coverage	2015 cut in SWDs funding disproportionally affect smaller HEIs in comparison to bigger ones Overreliance on private funding as a source of HEI income
USA	*Americans with Disabilities Act* (1990); *Rehabilitation Act* (1973)	Pell Grant Program	Assistive technology	-	2009 federal cuts; increased tuition fees
Canada	Canada Charter of Rights & the Canadian Charter of Rights and Freedoms (CCRF)	Canada States Grant for Services & Equipment	Assistive technology	No tuition, tools, general core requisition coverage	-
Australia	A Fair Chance for All (1990)	Disability Support Programme	Assistive technology, Sign language support provided	-	-
South Africa	National Plan for Higher Education, (2001), DOE (1997), DHET (2012)	NSFAS	Tuition, assistive technology	No sign language support, No personal support coverage	Decline in funding
India	Equal opportunities protection (1995), *Persons with Disabilities Act* (1995)	-	Assistive technology	-	Decline in funding

## Sampled countries’ funding trends

The inclusion of SWDs in the UK HEIs is currently facilitated by the *Equality Act*, 2010 (c. 15). Chapter 2 of the Act prohibits discrimination against PWDs in all spheres of their lives including in higher education. Disability policies are not only aimed at widening access to higher education but also at promoting success in higher education for SWDs. Widening participation is conceptualised as ‘opening higher education up to people who might not traditionally have considered university while improving retention, because students from different backgrounds need different support to complete their courses successfully’ (Bourn 2007). To achieve these goals, the Higher Education Funding Council for England, for example, enjoins:

all higher education providers and stakeholders [*to*] take a broad view of widening participation to encompass a student’s entire lifecycle: preparing for and entering higher education, graduating successfully, and progressing to employment or postgraduate study. (Department for Business, Innovation and Skills 2014:9)

The UK’s higher education funding model for SWDs draws mainly on the principle of ‘equality in access to education’ (Tumelty 2007) irrespective of ‘a person’s age, ethnicity, gender, disability and/or social background’ (Department for Business, Innovation and Skills 2014:7). The inclusion of disabled students in higher education has been supported by a number of measures, including the non-means tested DSA grant which:

provides extra financial help if you have a disability or a specific learning difficulty like dyslexia. This is paid on top of the standard student finance package and does not have to be repaid. (Disabled World 2016)

Full-time, part-time and postgraduate students can apply for DSA. Premium Funding is a funding allocation to HEIs to facilitate access for SWDs (Research Briefing 2008:2). Through Student Opportunity funding, the HEFCE also provides funds to HEIs in recognition of the additional costs of recruiting, supporting and retaining SWDs (OECD Higher Education Programme IMHE 2014:20).

In the USA, Congress enacted Section 504 of the *Rehabilitation Act* of 1973 as a measure to prohibit ‘discrimination against otherwise qualified persons with disabilities in any program receiving federal funds’ (Wolanin & Steele 2004:53). Section 504 prohibits denial of admission to a person because of their disability (Wolanin & Steele 2004:34). With the passage of the *Rehabilitation Act* of 1973, which defined facilities that are ‘inaccessible to or unusable by handicapped persons’ to be a form of prohibited discrimination, incentives were put in place in the form of federal funding to HEIs to enable them to make their facilities such as lecture theatres accessible to SWDs (Wolanin & Steele 2004:34). The *Americans with Disabilities Act* of 1990 (ADA) played an important role in enhancing the inclusion of SWDs by prohibiting discrimination against these students on the grounds of disability.

Under the ADA, discrimination against SWDs in American HEIs in relation to recruitment and admissions, academic and athletic activities, student examinations and evaluations, housing, financial aid, counselling, and career planning and placement is unconditionally prohibited (Kalivoda & Higbee 1994:133). The ADA also provides for the accessibility of the built environment by requiring public and private HEIs that are recipients of state or federal funds to adopt ‘accessible design of public places and facilities for all people, making buildings and facilities easily accessible to people with disabilities’ (Wolanin & Steele 2004:54). The education of SWDs is financed by a federal student aid programme, known as the Pell Grant Program, which supports eligible full-time SWDs (Wolanin & Steele 2004:ix–x). These students can apply for bursaries, non-repayable grants, loans or state or federally funded allowances managed by individual universities (OECD 2011:54).

Canada has taken a human rights approach to fostering the inclusion of SWDs (Thomas 2012:58–59) as is reflected in the CHRA principle that all individuals, including PWDs, should have equal opportunities and by prohibiting discrimination on the basis of disability (UNESCO 2015:37). In particular, the Act prohibits the denial of education to PWDs on the grounds of a disability as both discriminatory and illegal (UNESCO 2015:37). Section 15 of the CHRA provides for ‘equality of all people under the law and protection of individuals against discrimination on the basis of disability’ (Roeher Institute 1996). The CCRF states that, ‘[e]very individual is equal before the law and has the right to equal protection and equal benefit of the law without discrimination and, in particular, without discrimination based on … mental or physical disability’ among other grounds (UNESCO 2015:37).

The Canada Student Grant for Services and Equipment for Students with Permanent Disabilities (CSGSESPD) is a non-repayable grant designed to help students overcome educational disability-related barriers that they may encounter while pursuing post-secondary training (Department of Workforce and Advanced Learning Student Financial Services (DWALSFS) 2016:2). Eligible students under this programme ‘may receive funding of up to $8000 to cover the costs of needed goods and/or services that are directly related to overcoming the educational barriers that the disability may present’ (DWALSFS 2016:2). Goods and/or services covered by the grant include, for instance, ‘tutors; specialised transportation (to and from school); note takers; interpreters; attendant care for studies; readers; alternate format; reimbursements for learning disability assessments; assistive technology’ (DWALSFS 2016:2). The Canadian Province of Ontario BSWD provides non-repayable financial assistance ‘to assist students in meeting additional costs of equipment and supplies related to their participation in post-secondary education, which the student must incur because of his or her disability’ (Ontario Human Rights Commission 2016).

It is worth noting that 1990 saw the Australian government target widening of access to HEIs for students from deprived socio-economic backgrounds (including SWDs) in its policy document *A Fair Chance for All* (Department of Employment, Education and Training (DEET) 1990). The stated rationale was:

to ensure that Australians from all groups [*including SWDs*] in society have the opportunity to participate successfully in higher education. This will be achieved by changing the balance of the [*university*] student population to reflect more closely the composition of society as a whole. (DEET 1990:8)

*A Fair Chance for All*’s aims were twofold: firstly, ‘to double the present commencing enrolments of people with disabilities by 1995’ (Department of Employment, Education and Training (DEET) 1990:40); and secondly, to ensure the participation with success of SWDs by making it ‘unlawful [for] the development or approval of curriculum that excludes people with disabilities from participation’ (Brett 2010:4–5). Subsequently, the DDA of 1992 was passed, which obliges the Australian government to support the participation of all PWDs at all levels of education including making it unlawful to exclude SWDs from universities on the grounds of disability (MacLean & Gannon 1997:217).

Following South Africa’s first democratic election in 1994, the policy context changed rapidly in support of increasing and broadening access to university study as one aspect of redressing past inequalities (Cloete 2002). This commitment to equity and access was reflected in policy documents of the time (DOE 1997; Ministry of Education 2001) and continues to be emphasised in more recent policy making (DHET 2012; National Planning Commission 2011).

During apartheid, South African black learners with disabilities were low on the priority list of the National Party government (Fagin 2011:7). The 1948 *Special Schools Act* (SSA) provided for a segregated education system, which categorised children with disabilities according to both race and disability (Muthukrishna & Schoeman 2000), which made it difficult for learners with disabilities to access HEIs. The post-apartheid African National Congress (ANC) government’s early policymakers developed several higher education policies aimed at ‘putting in place appropriate redress strategies for the past inequities of the apartheid era’ (Mapesela & Hay 2005:112) aimed at radical transformation of South Africa’s higher education environment (Badat 2010:2). ‘Transformation’, thus, became a shorthand term to encapsulate a variety of initiatives aimed at ‘removing barriers and providing access to higher education for Black students, disadvantaged groups, and women’ (Belyakov et al. 2009:1).

South Africa’s post-1994 higher education disability policies (see, e.g., the 1997 *White Paper 3 on Higher Education Transformation*; the 1997 *White Paper on an Integrated National Disability Strategy*; the 1997 *Higher Education Act*; the 2001 *National Plan for Higher Education* and the 2001 *Education White Paper 6, Special Needs Education: Building an Inclusive Education and Training System*) draw from the country’s constitution in their emphasis on the need to address the disadvantages that PWDs experienced in the past and continue to experience, and the need to prioritise funding of higher education opportunities for SWDs in the present. *Education White Paper 3* called for the establishment of a new funding mechanism to achieve the principles of equity and redress through the abolition of all forms of discrimination including on grounds of disability through empowerment measures, ‘including financial support to bring about equal opportunity for individuals and institutions’ (DOE 1997:7–8). The 1996 *Green Paper* proposed the implementation of ‘funding mechanisms that will embody the principles of affordability, sustainability and shared costs, as well as those of equity, redress, development, democratisation, effectiveness and efficiency’ (DOE 1996:6). The *National Plan for Higher Education* calls on HEIs, through their institutional plans and strategies, to commit themselves to increase access for people with special education needs (Ministry of Education 2001:41). These policies have been applauded as ‘the best in the world, meeting internationally acclaimed standards’ (see, e.g., Mapesela & Hay 2005:112).

In 1996, the NSFAS – a student loan scheme to fund needy but capable students in higher education was established (Cele & Menon 2006:43). National Student Financial Aid Scheme caters for SWDs with an NSFAS bursary scheme tailored to giving non-means tested financial support to SWDs to study at one of the country’s 23 public HEIs in South Africa (National Student Financial Aid Scheme 2012a:3). Through the NSFAS scheme the government:

intended to open opportunities in higher and further education and training and provide the necessary additional teaching and learning (curriculum) support for students to overcome any barriers to learning which have resulted from their disability. (NSFAS 2012a:3)

Drawing on United Nations (UN) reports, Thomas (2012:59) has singled out India as ‘ha[ving] the best disability policies among the developing countries’. India has committed considerable financial resources to the implementation of inclusive education at primary, secondary and higher education levels (Thomas 2005). The education of SWDs is regulated by the 1995 *Equal Opportunities, Protection of Rights and Full Participation (PWD) Act*, which prohibits discrimination in every sphere on the grounds of disability (Thomas 2005).

## Impact of disability policies on increasing the numbers of students with disabilities in higher education

The general consensus within the reviewed literature in sampled countries is that these sampled countries’ funding mechanisms have positively resulted in a steady increase of SWDs in HEIs. In the UK context, for instance, it is worth noting that since 1997, the HEFCE has been instrumental in providing specific funding to HEIs to assist with the costs of delivering quality education for SWDs (Department for Business, Innovation and Skills 2014:64). In 2006–2007, for instance, it is estimated that the Funding Council allocated GB£13 million to HEIs to help in meeting additional costs in recruiting and retaining SWDs who are recipients of the DSA (National Audit Office 2008:6). In 2012–2013 alone:

nearly £150 million was spent on DSA for some 60 000 students providing a range of specialist equipment, such as computer software for those with dyslexia, as well as modifications to accommodation and extra support to disabled students. (Times Higher Education 2015)

In 2013–2014, £15 million was delivered by the HEFCE through its mainstream disability allocation, that is, around 4.5% of the total 2013–2014 HEFCE targeted allocations for widening participation and improving retention, which was £332 million (Department for Business, Innovation and Skills 2014:64). Disability funding has resulted in most UK universities successfully supporting the inclusion of SWDs particularly those which have adopted ‘an inclusive model that seeks to ensure all aspects of the institutional offer are accessible to disabled students’ (Department for Business, Innovation and Skills 2014:64). Against this background, Bourn (2007) has attributed the increase in the number of students declaring a disability entering higher education by over two-thirds between 2000–2001 and 2005–2006, from 82 000 to 138 000, to the DSA as these students were DSAs recipients.

Research in the USA has shown that as a result of supportive funding mechanisms the numbers of SWDs in HEIs tripled over the past 20 years (Myers 2008). Pell Grant, for instance, awards amounted to some EUR 15 million in 2009 (OECD 2011:54). The passage of anti-discriminatory legislation in the 1990s in Canada facilitated inclusive education, and changes in the attitudes of Canadian society. The number of SWDs attending post-secondary education increased steadily. For instance, the OECD estimated that in Canada’s Ontario province SWDs’ enrolment rose from 1668 in 1989–1990 to 6883 in 2000–2001 (OECD 2003). While in 1995 a mere 0.25% of SWDs registered to receive disability-related services in 47 Canadian universities, in 2003 this percentage had increased to 5.67% (Fichten et al. 2003). From 2001 to 2013, education rates of Canadian individuals with disabilities increased by 12.3%, with 74.6% of working-age adults with disabilities obtaining a high school diploma or higher educational certification (HRSD 2013).

Because of expansive financial support provided to SWDs, Australia experienced increased enrolments of SWDs in higher education, with students with learning disabilities being the largest enrolment group (Noble 1993). In 2012, nearly 6000 SWDs accessed equipment and educational support made available by Australian universities using DSP funds (OECD Higher Education Programme IMHE 2014:4). In 2014 under the DSP, $6.9 million was made available to universities for this purpose. The introduction of the NSFAS scheme in South Africa has translated into many visible changes in the sector as evidenced by the increase in the number of SWDs enrolling in HEIs (Wilson-Strydom 2015:144). In the 2011 academic year alone, government earmarked R76.8 million ‘to increase the funding available to SWDs and learners with special needs’ (University World News 2011). According to information recorded in the Higher Education Management Information System (HEMIS) this allocation of R76.8 million subsequently saw an increase in the ‘number of enrolled students with disabilities from 5856 in 2011 to 7110 in 2013’ (Hammond 2015). Through NSFAS, R45.5 million in bursaries went to 1368 SWDs in 2012 and R69.9 million benefited 1383 SWDs in 2014 (Hammond 2015).

India has also witnessed a significant increase in the number of SWDs in HEIs, which many have attributed to the policy of inclusive education backed by the availability of financial support (Shenoy 2011:5). These policies include the *Persons with Disabilities Act* (1995), which ‘came into action by bringing into sharp focus the state’s responsibility to empower the disabled with equal opportunities, protection of rights and full participation in the country’s development process’ (The Tenth Five Year Plan 2002–2007:471). The Act obliges HEIs to provide equal access and proportionate opportunities to excel in education at all levels (Pillai 2012:15). Proponents have applauded the Act for its adoption of an affirmative action approach (Kumar 2012). Section 39 aims to achieve the goal of increasing access to education for SWDs through mandating all public HEIs that are government aided to reserve a 3% quota for SWDs (Krishnan 2012:6).

Students with disabilities enrolling in Indian colleges are exempted from paying fees if they are not able to obtain financial assistance and each university receives a one-time grant of 1 000 000 Rupees as an incentive for enrolling the maximum number of SWDs (Jameel 2011:15). The HEPSN scheme established by the Indian government provides grants to support the setting up of Disability Units in universities on condition that the applying university has a sufficient number of students with documented disabilities (Krishnan 2012:6). Angela Kohama has argued that India’s *Persons with Disabilities Act* ‘functioned as a catalyst for several other development projects around inclusion and disability’ (Kohama 2012:21–22). In addition to the HEPSN scheme, provisions have been made for ‘incentives such as scholarships, both domestic and overseas to the SWDs with good academic records in higher education’ (Jameel 2011:6).

## Impact of sampled countries’ funding mechanisms

Global research on retention has pointed to the availability of adequate financial aid as one of the most important determining factors when it comes to low-income and minority students enrolling in, and persisting to, degree completion in HEIs (Swail 2004:9). As a number of studies have shown, funding is critical to the challenge of increasing enrollments of SWDs in HEIs (see, e.g., Jameel 2011:15; Katsui 2009; Research Briefing 2008:2). Prior to the 1970s, many SWDs both in developed and developing countries found it difficult to access higher education (HE) unless they had considerable personal means at their disposal (Kamalam et al. 2004:332). Availability of funding resulted in ‘disabled people increasingly hav[ing] access to educational opportunities that were not available to them in the past’ (Foley & Ferri 2012:192).

The growing proportion of SWDs enrolled in OECD countries’ higher education has been attributed to the introduction of financial incentives resources provided to HEIs (OECD 2003), which ‘offset the additional costs that the presence of a student with special education needs may represent for the institution’ (OECD 2011:55). It has been seen that the sample countries’ response to prioritising increasing access to HEIs for SWDs gained momentum in the early 1990s following the 1998 UNESCO World Conference on Higher Education leading to global international calls for greater ‘equality of access’ (UNESCO 1998).

Although all the sampled countries have supportive disability policies, however, in practice these countries are struggling to meet the goals outlined in their policies. As for the UK, an overall increase in reliance on private funding as a source of HEIs’ income (European Union 2014:28) and cuts to DSAs have resulted in SWDs being in debt as well as care cuts resulting in SWDs not, for example, having access to a personal assistant to make it physically possible to get to lectures (Ryan 2017). Cuts to DSAs were first proposed in 2014 when David Willetts, the then Universities Minister, announced a change in approach to the funding of computer equipment, software and consumable items through DSAs (Dunn 2016). In September 2015, the UK government officially confirmed that it would reduce direct public support for SWDs by making £30 million in cuts to DSA funding for SWDs in higher education (Dunn 2016). Willets urged universities to provide the support that was once provided by the DSA but without any funding allocated to cover the cost of this support.

In other words, without allocated funding universities are expected to pick up the tab for items that the DSA used to cover including, for instance, ‘funding the provision of non-medical support staff, such as scribes, note takers, readers, proof-readers and sign language interpreters’ (Dunn 2016). A particular worry is that these cuts to the DSA will disproportionately affect smaller institutions, which may lack the resources to fund adequate support for SWDs. The National Union of Students (NUS) protested against the government’s intentions to cut funding when it was first announced in June 2014 and branded them as ‘arrogant and out of touch’ (Times Higher Education 2015) with recent figures showing that approximately 70 000 students would be affected (Dunn 2016). The announced changes to the DSA will undoubtedly jeopardise access and success for SWDs (Government United Kingdom 2016). Given that one of the core services being targeted is funding for assistive technology, such as laptops with specialist digital voice recording, it has been argued that students with a specific learning disability like dyslexia will be notably affected by this move as these students rely most on a range of specialist equipment (Times Higher Education 2015).

However, the US recession of 2007 to 2009 saw federal cuts and some universities increasing their tuition fees (Camera 2016). The US HEIs face the challenge of ‘programs that should cooperate and coordinate to the benefit of SWDs often [*competing*] with each other to the detriment of these students’ (Moore 2003:9–11). Growing numbers of SWDs pose a huge challenge primarily to HEIs, whose federal funding campus-based programmes such as Perkins Loans, Supplemental Educational Opportunity Grants and College Work Study are inadequate because the demand for funds far exceeds the available funds (Wolanin & Steele 2004:60). Despite the obligations imposed by the *Rehabilitation Act* of 1973 upon HEIs in relation to RAs for SWDs, in the context of budget constraints, many institutions object strongly to the cost of compliance (Scotch 2001:125).

In the Canadian context, there are limitations – while in terms of the CSGSESPD grant recipients receive up to $8000 to cover the costs of academic-oriented needs, ‘the grant cannot be used to cover the cost of tuition, books, or items that are considered general requirements for the program’ (DWALSFS 2016:1), which means that these students have to pay out of pocket for these costs. Similarly, financial assistance provided under the BSWD is restricted to purchasing specialised equipment and services required for participating in HE studies and not RA related to tuition, books and housing expenses, which are the obligation of HEIs (Ontario Human Rights Commission 2016). In 2016, although the federal budget allocated $118.2 million over 2 years for students with a disability, the funding targeted schools and saw cuts of $152.2 million over 4 years to the Higher Education Participation Program, which funds Canadian ‘universities to bring in students from the lowest socio-economic levels [including SWDs]’ (Ryan 2017).

In Australia, the DDA regulates how RAs^2^ should be applied in higher education environments and places ‘a duty on institutions to make reasonable and anticipatory adjustments for disabled students in relation to teaching, learning and assessment’ (Research Briefing 2008:2). Services supported by the DSP include production of course materials and lecture notes in Braille, assistance with examinations and other assessment tasks and ‘purchase of adaptive software and/or adaptive computer equipment such as adaptive keyboards, mouse, screens, etc’. (OECD Higher Education Programme IMHE 2014:4). The passage of Australia’s DDA Standards for Education of 2005 saw the introduction of equity standards for the elimination of discrimination against PWDs in terms of their access to services and education (DSD, DWCPD & UNICEF 2012:99).

In South Africa, public HEIs’ budgetary constraints dating back to the late 1990s have inhibited the achievement of these aspirations: ‘university funding declined in terms of the proportion of total state finance committed to higher education from 4% in 1999 to 2.5% in 2007 forcing universities to raise tuition fees sharply’ (Shrivastava & Shrivastava 2014:815). Although the ‘NSFAS will aid well over 400‚000 students on a budget of R15-billion this year, [2017]’ (Collins 2017), nothing has been said yet as to whether the government will make similar increases to funding for SWDs. National Student Financial Aid Schemes SWD recipients increased from 701 in 2004 to 1112 in 2007 and dropped to 649 in 2009 (NSFAS 2012b). Delays of NSFAS bursaries reaching SWDs negatively resulted in many of these students dropping out (DHET 2009:24). South African HEIs’ Disability Units have an obligation to provide sign language interpreters to help with the inclusion of students who are deaf, but this is a costly option resulting in institutions resorting instead to purchasing assistive devices, which is not the optimal outcome for these students.

South African students who are deaf are currently facing the challenge of the unavailability of professional South African sign language interpreters, which has forced these students to rely on ‘fake’ sign language interpreters who also ‘take advantage of Deaf students, because they are desperately in need of access to education and will not complain about the lack of good sign language skills from the interpreters’ (DHET 2015:10). A 2011 study that sampled Disability Unit staff from 15 South African HEIs (FOTIM 2011) highlighted the fact that students who are deaf and hearing impaired who were NSFAS funding recipients expressed concerns over insufficiency of funding which forced them to resort to additional sources such as disability grants and parents’ contributions as a way of supplementing their funding (FOTIM 2011:83). The study also found that disability bursaries and scholarships often do not cover personal needs such as caregivers, for example, for quadriplegics (FOTIM 2011:84).

In India, research indicates that advances in access and equity are threatened by steadily declining financing of HEIs by the Indian government since the mid-1990s, which has resulted in higher education increasingly being ‘financed by non-government money, including household expenditures, fees, student loans, and voluntary contributions’ (Prakash 2007 cited in Yang & McCall 2014:28). These challenges which continue to be faced by the sampled countries clearly shed light on the fact that the mere provision of funding does not guarantee success once SWDs are enrolled (Belyakov et al. 2009:24). As defined by Belyakov et al., ‘access with success goes a step further, defining true access as completion of a degree or certificate program that prepares one for a vocation’ (Belyakov et al. 2009:1–3). The provision of funding alone does not result in the realisation of meaningful inclusive education (Ferguson 2008). Access and presence in ‘mainstream’ classrooms and schools are a necessary – but clearly not sufficient – step towards inclusive education for SWDs.

## Discussion

As Bowen (2009) points out, document analysis can provide a means to track change and historical processes. Here, we track a process of the countries selected for review putting in place funding mechanisms and policies, leading to an increase in the participation rate in higher education of SWDs, followed by subsequent funding cutbacks and the privatisation and marketisation of higher education leading to the erosion of some of these gains. In the context of budget constraints, we see a pattern of HEIs falling back to a position of minimal accommodation for SWDs and an approach of minimal legal compliance rather than seeing their responsibility as extending to the fullest possible realisation of the equal right to access and success of SWDs.

A comparative analysis of the sampled countries’ funding mechanisms shows that these countries have had considerable successes in relation to increasing access to higher education for SWDs. However, declining government support for education has led to a massive rise in the for-profit education industry, which has influenced the way in which universities set their funding priorities (Kenway et al. 1993:2) and has resulted in ‘an ideological shift towards higher education as a private rather than a public good’ (Meek 2000:24). In the words of Newman and Jahdib (2009:1), this has led to ‘a paradigm shift’ in the form of the ‘so-called marketisation of education’. According to De Jager and Gbadamosi (2010:254), the marketisation of higher education has resulted in ‘a more competitive educational environment’ in which universities compete with one another ‘for students, resources and prestige’ (Meek 2000:23) and in which students are framed as consumers of education (Molesworth et al. 2009:277). These shifts have taken their toll on social inclusion initiatives. This is not a phenomenon of the developed world alone. Government funding cuts lead to rising operational costs, hiring freezes and large classes (Shrivastava & Shrivastava 2014:815) – all of which have the potential to negatively impact the inclusion of SWDs.

While all the countries surveyed here are signatories to the UNCRPD of 2006 (DSD et al. 2012:19), the economic context impinges on their ability to fulfil their obligations in terms of the Convention not only with respect to the provision of funding to afford access to higher education for SWDs but also to cover expenses such as ‘auxiliary aids to provide accommodations to students with disabilities’ to achieve ‘a level playing field’ in higher education (Wolanin & Steele 2004:58).

Reliance on private rather than public funding has seen most universities breaching these obligations and serving market-driven rather public interests in relation to human health, safety or well-being (Côté-Boucher 2010). The consequences have proved detrimental to SWDs as their HEIs lose autonomy to private funders. Elaborating on this, Jung (2003) argues that this has resulted in universities fulfilling only their most ‘minimal’ legal and moral obligations to provide RAs to SWDs – principally disability accommodations necessary for their successful participation in the classroom.

By relying on private funding sources institutions are limited to the restrictions that come with donations – with private funders, for example, regarding personnel costs as the institution’s responsibility and preferring to pay only for equipment rather than sign language interpreters (Howell 2005:10).

This exclusion has, according to Eleweke and Rodda (2002:115–116), created an unfavourable situation where upon gaining admission these students ‘are [left] on their own as they receive no special support to help them on their courses’. The rationale behind selective inclusion is the marketised framing of these students as difficult to accommodate as a result of needing ‘too much’ specialist assistance (Singal 2005:6). Under this selective inclusion approach, students who are deaf are being poorly served especially in countries like South Africa as NSFAS guidelines ‘do not fund human support (scribes, sign-language interpreters and note takers, etc.)’ (FOTIM 2011:137). The same holds true for some Indian HEIs such as Adarsh College in Chamarajpet, Bengaluru University and Delhi University where the absence of sign language interpreters has forced some students who are deaf to pay out of pocket themselves for lessons with private interpreters at exorbitant rates (Krishnan 2012:7). In contrast, Australia stands out among the sampled countries as having been able to maintain good practices despite the challenges of budgetary constraints as it has responded positively to the increasing enrolment levels of deaf and hard of hearing in its HEIs by employing more and more interpreters (Knuckey et al. 2001; see also OECD Higher Education Programme IMHE 2014:4).

Following Howell, achieving genuine inclusive education for SWDs does not merely end with dismantling physical access barriers to HEIs, but most importantly through also putting in place mechanisms that can provide additional support to those SWDs who may require it (Howell 2005:13). However, evidence from our findings indicates that this has not come to reality as most HEIs seem to focus mainly on achieving physical access for SWDs, thus leaving their academic success in jeopardy. As one Lancaster University DSA recipient who has autism commented:

just because I made it to university, does not mean I’ll cope without support … Without DSA, the trivial things would become impossible for me – this also applies to many future disabled students, who are being ignored by the government. (Times Higher Education 2015)

Students with disabilities must meet these additional costs in the course of coping with their disabilities, but often their incomes are lower than those of their non-disabled peers, which means that their dependence on state funding is higher, yet ‘their opportunities have been diminished by the inadequate levels of financial aid, particularly grants’ tailored for low-income SWDs (Wolanin & Steele 2004:ix–x). Funding cutbacks have meant that HEIs do not have enough funds available to meet all the needs of SWDs, resulting in SWDs themselves having to bear some of these costs out of pocket (Wolanin & Steele 2004:58). Similarly, ‘the availability of state and institutional financial aid funds also is limited by either award limits or an excess of demand compared to funding’ (Wolanin & Steele 2004:60).

In both global North and global South, PWDs are considered among the world’s most vulnerable and least empowered groups (Khasnabis et al. 2010:11). This vulnerability of PWDs has been attributed to high levels of poverty, which has resulted in this group’s experiencing worse edu-cational and labour market outcomes in comparison to their non-disabled peers (World Health Organization (WHO) & World Bank 2011:39). The findings of this study show that the funding mechanisms of sampled countries still deny SWDs the capabilities to escape the poverty cycle by empowering themselves through acquiring tertiary education, which enhances their prospect of employment. This is particularly true of SWDs who are reliant on assistive technology, which is not covered in most of the sampled countries’ funding mechanisms. This becomes a violation of inclusive education as provided in the UNCRPD, which imposes a responsibility on state parties to honour their responsibility to promoting and ensuring the availability and access to assistive technologies for SWDs if they are to participate fully in the classroom setting (United Nations 2006:6).

We critique the incentivisation of disability inclusion which is prevalent in the USA (Wolanin & Steele 2004:34), Canada (OECD Higher Education Programme IMHE 2014:4) and India (Jameel 2011:15) where funding to these countries has been allocated based on a number of SWDs enrolled annually. Although we acknowledge that in the wake of serious resource constraints HEIs will need funding and resources should be made available to assist them in driving the disability agenda (FOTIM 2011:14), we argue that this approach will continue to benefit bigger HEIs in cities at the cost of smaller HEIs, especially those in under-resourced rural settings.

In India, for instance, given the abundance of assistive technology, the Indian Institute of Technology in Delhi has attracted many SWDs nationwide to come to study there (Joseph 2012:1), which means that this institution will always receive more funding than other Indian universities. Likewise, despite the fact that South Africa has leading universities which are internationally respected, because of the legacy of apartheid historically black universities continue to face severe financial, human, infrastructure and other resource constraints (Badat 2015). Unlike South African historically white institutions, some of these historically black universities still do not have Disability Units, which makes them unwelcoming to students with diverse disabilities. Seen from this perspective, we propose the need for the sampled countries to provide disability funding as per provided in their individual funding mechanisms as well as per the provisions of the UNCRPD and they should pay particular attention in prioritising the funding for smaller universities and universities of technology.

## Conclusion

The OECD has pointed to inadequate funding as one of the hindrances to the successful transition to tertiary education for SWDs (OECD 2011:69). Living with a disability entails expenses such as trips to doctors, therapists, counsellors and administrators (Wolanin & Steele 2004:61). While under all the sampled countries’ funding models, SWDs are entitled to funding to offset the extra costs of living with a disability or a specific learning difficulty (OECD 2011:55), the current financial context sees institutions facing challenges with the provision of adequate funding to cover the extra costs which are incurred by SWDs in the course of pursuing their studies in HEIs.

While the increase in numbers of SWDs enrolling in HEIs was, as we saw above, made possible by publicly funded grants, with declining public funding, there is a high possibility that numbers will once again fall. As access to grants and scholarships narrows, ‘low-income students with disabilities [who] generally have a greater need for financial aid than their peers without disabilities’ will be most affected (Wolanin & Steele 2004:63). Although the sample countries have widely embraced the practices of inclusive education as an ideal model for education (Maher 2009), these countries still have a long way to go with regard to adequately financially supporting SWDs in a manner which widens their opportunities to access, participate in, be retained in and succeed in HEIs. Because of funding challenges, it can be argued that inclusive education is being implemented narrowly in these countries in a way which perpetuates the exclusion and marginalisation of SWDs. As a result, the principle of inclusive education ‘increasing participation and reducing exclusion, in a way that effectively responds to the diverse needs of all learners’ (Kaur & Arora 2014:59) is routinely violated in practice.

Because of inadequate funding, we see many countries resorting to selective inclusion or what Ndlovu and Walton (2016:7) have referred to as an ‘impairment based approach’ in supporting SWDs in which only particular categories of disability are accommodated by specific institutions. Impairment-based approaches have often seen HEIs prioritising providing RAs to certain categories of SWDs at the cost of others. For example, students who are deaf fare differently compared to other students because of the shortage of, and cost involved in providing, fluent sign language interpreters. This violates the overarching goal of inclusive education which is to ensure the participation of *all* SWDs in quality education to develop the full potential of these students (Kaur & Arora 2014:59). The result of prioritising some groups of SWDs at the cost of others is a form of selective inclusion determined by cost rather than meeting individual needs regardless of cost.

## Footnotes

1 Guided by the ‘United Nations Convention on the Rights of Persons with Disabilities’ we approach ‘disability’ as ‘an evolving concept that results from the interaction between persons with impairments and attitudinal and environmental barriers that hinder their full participation in society on an equal basis with others’ (United Nations 2006:5).

2 Kraglund-Gauthier and colleagues have defined RAs as:
involv[ing] minor changes that assist a student’s functioning in the classroom by offering alternate ways of handling a task; for example, providing photocopied notes to a student with muscular dystrophy who has difficulty with writing. (Kraglund-Gauthier et al. 2014:2) Common forms of accommodations in the postsecondary context would, for example, include provisions such as ‘extended time on exams, alternative exam formats, and assistance with note-taking, study skills, and learning strategies’ (Lombardi et al. 2011:250).

## References

[CIT0001] Ainscow M., Booth T. & Dyson A., 2004, ‘Understanding and developing inclusive practices in schools: A collaborative action research network’, *International Journal of Inclusive Education* 8(2), 125–139. 10.1080/1360311032000158015

[CIT0002] Badat S., 2010, ‘The challenges of transformation in higher education and training institutions in South Africa’, Paper Commissioned by the Development Bank of Southern Africa, Johannesburg, 08 April 2010.

[CIT0003] Badat S., 2015, ‘Institutional combinations and the creation of a new higher education institutional landscape in post-1994 South Africa’, in Curaj A., Georghiou L., Harper J.C. & Egron-Polak E. (eds.), *Mergers and alliances in higher education*, pp. 175–201, Springer, Cham.

[CIT0004] Belyakov A., Cremonini L., Mfusi M.X. & Buck J.R., 2009, *The effect of transitions on access to higher education*, Institute for Higher Education Policy, Washington, DC, Issue Brief, pp. 1–28.

[CIT0005] Bourn J., 2007, *Staying the course: The retention of students in higher education, National Audit Office*, 26 July, viewed 09 October 2016, from https://www.nao.org.uk/report/staying-the-course-the-retention-of-students-in-higher-education/

[CIT0006] Bowen G.A., 2009, ‘Document analysis as a qualitative research method’, *Qualitative Research Journal* 9(2), 27–40. 10.3316/QRJ0902027

[CIT0007] Brett M., 2010, ‘Challenges in managing disability in higher education, illustrated by support strategies for deaf and hard of hearing students’, *The Open Rehabilitation Journal* 3, 4–8. 10.2174/1874943701003010004

[CIT0008] Camera L., 2016, ‘States are slacking on higher education spending’, *US News*, 07 January, viewed 03 June 2017, from http://www.usnews.com/news/articles/2016/01/07/states-spending-less-on-higher-education-today-than-before-recession.

[CIT0009] Cele N. & Meno K., 2006, ‘Social exclusion, access and the restructuring of higher education in South Africa’, *South African Journal of Higher Education* 20(3), 38–50.

[CIT0010] Cloete N., 2002, *South African higher education and social transformation*, Higher Education Digest, Autumn Issue, Southampton, United Kingdom.

[CIT0011] Collins F., 2017, ‘NSFAS to fund more than 400‚000 students this year’, *The Guardian*, 19 February, viewed 03 June 2017, from http://www.heraldlive.co.za/news/2017/02/19/nsfas-fund-400000-students-year/

[CIT0012] Côté-Boucher K., 2010, ‘Risky business? Border preclearance and the securing of economic life in North America’, in Braedley S. & Luxton M. (eds.), *Neoliberalism and everyday life*, pp 37–67, McGill-Queens’s University Press, Montreal, QC.

[CIT0013] Daugherty L. Miller T. Dossani R. & Clifford M., 2013, *Building the links between funding and quality in higher education: India’s challenge*, RAND Corporation, viewed 01 October 2016, from www.rand.org

[CIT0014] De Jager J. & Gbadamosi G., 2010, ‘Specific remedy for specific problem: Measuring service quality in South African higher education’, *Higher Education* 60, 251–267. 10.1007/s10734-009-9298-6

[CIT0015] Department for Business, Innovation and Skills, 2014, *National strategy for access and student success in higher education*, Higher Education Funding Council for England, London.

[CIT0016] Department of Education (DOE), 1996, *Green paper on higher education transformation*, Department of Education, Pretoria.

[CIT0017] Department of Education (DOE), 1997, *Education white paper 3: A programme for the transformation of higher education*, Department of Education, Pretoria.

[CIT0018] Department of Employment Education Training & National Board of Employment, Education and Training (DEET), 1990, *A fair chance for all: National and institutional planning for equity in higher education*, A discussion paper, Australian Government Publishing Service, Canberra.

[CIT0019] Department of Higher Education and Training (DHET), 2009, *Report of the Ministerial Committee on the Review of the National Student Financial Aid Scheme*, Government Printers, Pretoria.

[CIT0020] Department of Higher Education and Training (DHET), 2012, *Green paper for post-school education and training*, Government Printers, Pretoria.

[CIT0021] Department of Higher Education and Training (DHET), 2015, ‘Higher education and training shares opportunities for disabled learners’, *South African Government*, 27 July, viewed 09 October 2016, from http://www.gov.za/speeches/disability-no-barrier-learning-post-school-education-and-training-system-27-jul-2015-0000

[CIT0022] Department of Workforce and Advanced Learning Student Financial Services (DWALSFS), 2016, *Canada student grant for services and equipment for students with disabilities: Information and guidelines*, Prince Edward Island, Canada, viewed 09 October 2016, from http://www.gov.pe.ca/forms/pdf/2075.pdf

[CIT0023] Disabled World, 2016, ‘Disability loans grants and low income finance’, *Disabled World*, 26 April, viewed 01 October 2016, from http://www.disabled-world.com/disability/finance/

[CIT0024] DSD, DWCPD & UNICEF, 2012, *Children with disabilities in South Africa: A situation analysis: 2001–2011*, Department of Social Development/Department of Women, Children and People with Disabilities/UNICEF, Pretoria.

[CIT0025] Dunn A.R., 2016, ‘Disabled students like me rely on funding at university. Now it’s been cut’, *The Guardian*, 27 January, viewed 01 October 2016, from https://www.theguardian.com/education/2016/jan/27/disabled-students-like-me-rely-on-funding-at-university-now-its-been-cut

[CIT0026] European Union, 2014, *Do changes in cost-sharing have an impact on the behaviour of students and higher education institutions? Evidence from nine case studies*, Volume I: Comparative Report, Publications Office of the European Union, Luxembourg.

[CIT0027] Eleweke C.J. & Rodda M., 2002, ‘The challenge of enhancing inclusive education in developing countries’, *International Journal of Inclusive Education* 6(2), 113–126. 10.1080/13603110110067190

[CIT0028] Fagin J.M., 2011, ‘Global influences and resistance within: Inclusive practices and South Africa’s apartheid government’, Master’s thesis, Paper 549 School of Education, Loyola University Chicago.

[CIT0029] Ferguson D.L., 2008, ‘International trends in inclusive education: The continuing challenge to teach each one and everyone’, *European Journal of Special Needs Education* 23(2), 109–120. 10.1080/08856250801946236

[CIT0030] Fichten C.S., Asuncion J.V., Barile M., Robillard C., Fossey M.E. & Lamb D., 2003, ‘Canadian postsecondary students with disabilities: Where are they?’, *The Canadian Journal of Higher Education* 33(3), 71–114.

[CIT0031] Foley A. & Ferri B.A., 2012, ‘Technology for people, not disabilities: Ensuring access and inclusion’, *Journal of Research in Special Educational Needs* 12(4), 192–200. 10.1111/j.1471-3802.2011.01230.x

[CIT0032] Foundation of Tertiary Institutions of the Northern Metropolis (FOTIM), 2011, *Disability in higher education: Project report*, Ford Foundation, Johannesburg.

[CIT0033] Government United Kingdom, 2016, ‘Disabled Students’ Allowances (DSAs)’, Government United Kingdom, viewed 01 October 2016, from https://www.gov.uk/disabled-students-allowances-dsas/what-youll-get.

[CIT0034] Hammond S., 2015, ‘NSFAS support for students with disabilities shows results in numbers enrolled and graduating’, *Skills Universe*, 22 March, viewed 01 October 2016, from http://www.skills-universe.com/group/green-paper-for-post-school-education-training/forum/topics/nsfas-support-for-students-with-disabilities-shows-results-in-num

[CIT0035] Howell C., 2005, *Higher education monitor, South African Higher Education responses to students with disabilities: Equity of access and opportunity?* The Council on Higher Education, Pretoria.

[CIT0036] Human Resources and Skills Development Canada (HRSD), 2013, *Federal disability reference guide*, viewed 01 October 2016, from http://www.esdc.gc.ca/eng/disability/arc/reference_guide.shtml.

[CIT0037] Jameel S.S., 2011, ‘Disability in the context of higher education: Issues and concerns in India’, *Electronic Journal for Inclusive Education* 2(7), 1–22.

[CIT0038] Jung K., 2003, ‘Chronic illness and academic accommodation: Meeting disabled students’ “unique needs” and preserving the institutional order of the university’, *Journal of Sociology and Social Welfare* 30(1), 91–112.

[CIT0039] Joseph D., 2012, *‘Foreword’ in enabling access for persons with disabilities to higher education and workplace: Role of ICT and assistive technologies*, Ford Foundation, viewed 28 May 2018, from www.nevertheless.in.

[CIT0040] Kalivoda S. & Higbee J.L., 1994, ‘Implementing the Americans with disabilities act’, *Journal of Humanistic Education and Development* 32(3), 133–137. 10.1002/j.2164-4683.1994.tb00141.x

[CIT0041] Kamalam M., Annuncia S.F. & Rajan, 2004, ‘Special needs for persons with disabilities’ in higher education’, in Stanley S. (ed.), *Social problems in India*, pp. 331–338, Allied Publishers, Mumbai.

[CIT0042] Katsui H., 2009, ‘Hisayo in accessibility for all at higher education seminar’, *Disability-Uganda*, viewed 01 October 2016, from http://disability-uganda.blogspot.com/2009/05/hisayo-in-accessibility-for-all-at.html.

[CIT0043] Kaur J. & Arora B., 2014, ‘Inclusive education – An integrated approach’, *IMPACT: International Journal of Research in Humanities, Arts and Literature* 2(2), 59–64.

[CIT0044] Kenway J., Bigum C., Fitzclarence L. & Collier J., 1993, ‘Marketing education in the 1990s’, *The Australian Universities’ Review* 36(2), 2–6.

[CIT0045] Khasnabis C., Heinicke Motsch K., Achu K., Al Jubah K., Brodtkorb S., Chervin P. et al, 2010, *Community-based rehabilitation: CBR guidelines*, World Health Organization, Geneva. 26290927

[CIT0046] Knuckey J., Lawford L. & Kay J., 2001, *Information should be visual: New and emerging technologies and their application in the vet sector for students who are deaf and hard of hearing*, National Centre for Vocational Education Research, Australian National Training Authority, Leabrook, South Australia.

[CIT0047] Kohama A., 2012, ‘Inclusive education in India: A country in transition’, Unpublished Undergraduate Honours thesis, Department of International Studies, University of Oregon.

[CIT0048] Kraglund-Gauthier W.L., Young D.C. & Kell E., 2014, ‘Teaching students with disabilities in post-secondary landscapes: Navigating elements of inclusion, differentiation, universal design for learning, and technology’, *Teaching Students with Disabilities* 7(3), 1–9.

[CIT0049] Krishnan R.T., 2012, ‘IIM Bangalore’s experience on providing equal opportunities’, in *Enabling access for persons with disabilities to higher education and workplace: Role of ICT and Assistive Technologies*, Ford Foundation, viewed October 2016, from www.nevertheless.

[CIT0050] Kumar A., 2012, ‘Making the disabled, differently-abled access higher education and employment’, in *Enabling access for persons with disabilities to higher education and workplace: Role of ICT and assistive technologies*, Ford Foundation, viewed 01 October 2016, from www.nevertheless.

[CIT0051] Lombardi A.R., Murray C. & Gerdes H., 2011, ‘College faculty and inclusive instruction: Self-reported attitudes and actions pertaining to universal design’, *Journal of Diversity in Higher Education* 4(4), 250–261. 10.1037/a0024961

[CIT0052] MacLean D. & Gannon P.M., 1997, ‘The emotionally affected university student: Support from the university community’, *International Journal of Disability, Development and Education* 44(3), 217–228. 10.1080/0156655970440304

[CIT0053] Maher M., 2009, ‘Information and advocacy: Forgotten components in the strategies for achieving inclusive education in South Africa?’, *Africa Education Review* 6(1), 19–36. 10.1080/18146620902857251

[CIT0054] Mapesela M. & Hay H.R., 2005, ‘Through the magnifying glass: A descriptive theoretical analysis of the possible impact of the South African higher education policies on academic staff and their job satisfaction’, *Higher Education* 50, 111–128. 10.1007/s10734-004-6358-9

[CIT0055] McGrath C.H., Henham M.L., Corbett A., Durazzi N., Frearson M., Janta B. et al, 2014, *Higher education entrance qualifications and exams in Europe: A comparison*, Policy Department B: Structural and Cohesion Policies, European Parliament’s Committee on Culture and Education, European Parliament, Brussels.

[CIT0056] Meek L.V., 2000, ‘Diversity and marketisation of higher education: Incompatible concepts?’, *Higher Education Policy* 13, 23–39. 10.1016/S0952-8733(99)00030-6

[CIT0057] Ministry of Education, 2001, *National plan for higher education*, South African Ministry of Education, Pretoria.

[CIT0058] Muthukrishna N. & Schoeman M., 2000, ‘From “special needs” to “quality education for all”: A participatory, problem-centered approach to policy development in South Africa’, *International Journal of Inclusive Education* 4(4), 315–335. 10.1080/13603110050168023

[CIT0059] Molesworth M., Nixon E. & Scullion R., 2009, ‘Having, being and higher education: The marketisation of the university and the transformation of the student into consumer’, *Teaching in Higher Education* 14(3), 277–287. 10.1080/13562510902898841

[CIT0060] Moore R., 2003, *Students with disabilities face financial aid barriers: College and graduate students share their stories and policy recommendations*, National Council on Disability, Washington, DC.

[CIT0061] Myers K., 2008, ‘Infusing universal instructional design into student personnel graduate programs’, in Higbee J. & Goff E. (eds.), *Pedagogy and student services for institutional transformation: Implementing universal design in higher education*, pp. 291–304, Center for Research on Developmental Education and Urban Literacy, University of Minnesota, Minneapolis, MN.

[CIT0062] National Audit Office, 2008, *Widening participation in higher education*, Report by the Comptroller and Auditor General, London.

[CIT0063] National Planning Commission (NPC), 2011, *National development plan*, Vision 2030, National Planning Commission, Office of the President, Pretoria, South Africa.

[CIT0064] National Student Financial Aid Scheme (NSFAS), 2012a, *Annual report 2012*, NSFAS, Cape Town.

[CIT0065] National Student Financial Aid Scheme (NSFAS), 2012b, *Number of students with disabilities assisted*, NSFAS, Cape Town, viewed 06 November 2017, from http://www.nsfas.org.za/profile-statistics.htm.

[CIT0066] Ndlovu S. & Walton E., 2016, ‘Preparation of students with disabilities to graduate into professions in the South African context of higher learning: Obstacles and opportunities’, *African Journal of Disability* 5(1), 1–8. 10.4102/ajod.v5i1.150 PMC5433445 28730040

[CIT0067] Newman S. & Jahdib K., 2009, ‘Marketisation of education: Marketing, rhetoric and reality’, *Journal of Further and Higher Education* 33(1), 1–11. 10.1080/03098770802638226

[CIT0068] Noble A., 1993, ‘Students with learning disabilities’, *Pathways: Post-Secondary Education Network Newsletter*, 04 May, pp. 14–15.

[CIT0069] OECD Higher Education Programme IMHE, 2014, *Fostering equity in higher education compendium of practical case studies: Fostering inclusion of disadvantaged students*, OECD Publishing, Paris.

[CIT0070] Ontario Human Rights Commission, 2016, *The opportunity to succeed: Achieving barrier-free education for students with disabilities*, viewed 01 October 2016, from http://www.ohrc.on.ca/en/opportunity-succeed-achieving-barrier-free-education-students-disabilities/post-secondary-education.

[CIT0071] Organisation for Economic Co-operation and Development (OECD), 2003, *Diversity, inclusion and equity: Insights from special needs education*, Education Policy Analysis, OECD Publishing, Paris.

[CIT0072] Organisation for Economic Co-operation and Development (OECD), 2011, *Inclusion of students with disabilities in higher education and employment*, Education and Training Policy, OECD Publishing, Paris.

[CIT0073] Pillai P.R., 2012, AN UNPHIL STRUGGLE? ‘Building inclusive libraries in India for students who are visually impaired’, in *Enabling access for persons with disabilities to higher education and workplace: Role of ICT and Assistive Technologies*, Ford Foundation, viewed September 2016, from www.nevertheless.

[CIT0074] Prakash V., 2007, ‘Trends in growth and financing of higher education in India’, *Economic and Political Weekly* 42(31), 3249–3258.

[CIT0075] Research Briefing, 2008, *Disabled students in higher education: Experiences and outcomes: Teaching and learning*, TLRP, London, vol. 46, pp. 1–4, viewed 04 September 2016, from http://www.tlrp.org/pub/documents/Fuller%20RB%2046%20FINAL.pdf.

[CIT0076] Roeher Institute, 1996, *Disability, community and society: Exploring the links*, North York, Ontario.

[CIT0077] Ryan F., 2017, ‘When will our politics start to address young disabled people?’, *The Guardian*, 15 June, viewed 03 June 2017, from https://www.theguardian.com/commentisfree/2017/jun/15/labour-young-disabled-people-vote-too?CMP=share_btn_link.

[CIT0078] Scotch R.K., 2001, *From goodwill to civil rights: Transforming federal disability policy*, Temple University Press, Philadelphia, PA.

[CIT0079] Shenoy M., 2011, *Persons with disability and the India labour market: Challenges and opportunities*, International Labour Organization (ILO): ILO Regional Office for Asia and the Pacific, Bangkok.

[CIT0080] Shrivastava M. & Shrivastava S., 2014, ‘Political economy of higher education: Comparing South Africa to trends in the world’, *Higher Education* 67, 809–822. 10.1007/s10734-013-9709-6

[CIT0081] Simons M. & Masschelein J., 2005, ‘Inclusive education for exclusive pupils: A critical analysis of the government of the exceptional’, in Tremain S. (ed.), *Foucault and the government of disability*, The University of Michigan Press, Ann Arbor, MI.

[CIT0082] Singal N., 2005, ‘Responding to difference: Policies to support ‘inclusive education’ in India’, Paper presented at the Inclusive and Supportive Education Congress, 01–04 August, University of Strathclyde, Glasgow.

[CIT0083] Swail W.S., 2004, *The art of student retention: A handbook for practitioners and administrators*, Texas Higher Education Coordinating Board, 20th Annual Recruitment and Retention Conference, Educational Policy Institute, Austin, TX, viewed 26 October 2016, from www.educationalpolicy.org.

[CIT0084] The Tenth Five Year Plan, 2002–2007, *Sectoral policies and programmes*, Planning Commission, Government of India, New Delhi.

[CIT0085] Thomas P., 2005, *Mainstreaming disability in development: India country report*, Disability Knowledge and Research, London, viewed 14 August 2016, from http://disabilitykar.net/research/polindia.html.

[CIT0086] Thomas A.S., 2012, ‘Disability through a human rights paradigm’, in *Enabling access for persons with disabilities to higher education and workplace: Role of ICT and Assistive Technologies*, Ford Foundation, viewed 09 September 2016, from www.nevertheless.

[CIT0087] Times Higher Education, 2015, ‘DSA cuts plans face high court scrutiny’, *Times Higher Education*, 03 May, viewed 01 October 2016, from https://www.timeshighereducation.com/news/dsa-cuts-plans-face-high-court-scrutiny/2018892.article.

[CIT0088] Tumelty G., 2007, ‘Equal access to education means an equal society’, *The Guardian*, 18 April, viewed 24 September 2016, from http://www.theguardian.com/education/mortarboard/2007/apr/18/gemmatumelty.

[CIT0089] UNESCO, 1994, *The salamanca statement and framework for action on special needs education*, UNESCO Publishing, Paris.

[CIT0090] UNESCO, 1998, *The world conference on higher education in the twenty-first century: Vision and action*, UNESCO Publishing, Paris, 05–09 October.

[CIT0091] UNESCO, 2015, *The right to education for persons with disabilities: Overview of the measures supporting the right to education for persons with disabilities reported on by member states*, Monitoring of the Implementation of the Convention and Recommendation against Discrimination in Education (8th Consultation) United Nations Educational, Scientific and Cultural Organisation, UNESCO, Paris.

[CIT0092] United Nations, 2006, *Convention for the rights of persons with disabilities*, United Nations, New York.

[CIT0093] University World News, 2011, ‘South Africa: Fund rescues non-fee-paying graduates’, *University World News*, 30 October, viewed 03 February 2018, from http://www.universityworldnews.com/article.php?story=20111029081324316.

[CIT0094] Wolanin T.R. & Steele P.E., 2004, *Higher education opportunities for students with disabilities: A primier for policy makers*, The Institute for Higher Education Policy, Washington, DC.

[CIT0095] World Health Organization (WHO) & World Bank, 2011, *World report on disability*, World Health Organization, Geneva, viewed 10 June 2018, from http://whqlibdoc.who.int/publications/2011/9789240685215_eng.pdf.

[CIT0096] Wilson-Strydom M., 2015, ‘University access for social justice: A capabilities perspective’, *Higher Education* 69, 143–155. 10.1007/s10734-014-9766-5

[CIT0097] Yang L. & McCall B., 2014, ‘World education finance policies and higher education access: A statistical analysis of world development indicators for 86 countries’, *International Journal of Educational Development* 35, 25–36. 10.1016/j.ijedudev.2012.11.002

